# Drivers of disperser immigration into cooperatively breeding carnivore groups

**DOI:** 10.1093/beheco/arag013

**Published:** 2026-02-10

**Authors:** Kathleen M Petersen, David E Ausband

**Affiliations:** Idaho Cooperative Fish and Wildlife Research Unit, University of Idaho, 875 Perimeter Drive, MS 1141, Moscow, ID 83844, United States; U.S. Geological Survey, Idaho Cooperative Fish and Wildlife Research Unit, University of Idaho, 875 Perimeter Drive, MS 1141, Moscow, ID 83844, United States

**Keywords:** cooperative breeding, gray wolf, dispersal, harvest, immigration, settlement

## Abstract

Dispersal is a fundamental process that shapes social groups by affecting genetic diversity, group composition, and social dynamics through immigration and subsequent settlement. In group-living animals, dispersal involves more than just leaving 1 group and arriving at another because dispersers also need to be accepted at an established group for successful dispersal to occur. Understanding how and why new individuals integrate into established social groups remains a key question, particularly when the benefits to existing members are unclear. This question persists in part because the ecological and social conditions that shape disperser settlement remain poorly understood. We leveraged an existing harvest regime and examined 18 years of life-history data from a wild population of cooperatively breeding gray wolves (*Canis lupus*) to understand immigration dynamics of group-living. Specifically, we tested how social and environmental conditions within groups predicted the likelihood that a disperser successfully immigrated into a group, analyzing how breeder turnover, annual harvest, group size, and genetic relatedness influenced that decision. Turnover of breeding males had the strongest effect on the probability of disperser settlement, suggesting that the loss of key social roles may create opportunities for new individuals to join groups. We also found an interaction between group size and harvest. By quantifying conditions that shape immigrant settlement, we highlight a mechanism influencing the stability and structure of cooperatively breeding groups. Unlike studies focused on individual dispersal decisions, our research highlights how variation in ecological and social conditions shape settlement into groups by dispersers.

## Teaser

When a disperser leaves home, what shapes entry into a new group? Group-living animals do not welcome every outsider. Using long-term genetic data from gray wolves, we found that social change, like the loss of the breeding male, can open a path for immigration. Settlement in groups is not just about timing but also group size and human impact.

## Introduction

Dispersal is a widespread and ecologically fundamental process that influences population dynamics, genetic diversity, and species distribution (Clobert et al. 2012). It consists of 3 distinct stages: emigration, transience, and immigration (Bowler and Benton 2005). During the immigration stage in particular, species settle due to a combination of ecological, social, and individual factors, such as resource and territory availability, the presence of conspecifics, and social cues (Clobert et al. 2012). Immigration can be highly variable and influenced by the availability of suitable habitats and social opportunities, like vacant territories and potential mates (Boyd and Pletscher 1999; Morales-González et al. 2021). Furthermore, successful immigration, or settlement, can directly increase population size in settlement territories (Espenshade et al. 1982), serve as sources of genetic connectivity, reduce inbreeding among distinct populations (Wright 1949; Koenig and Pitelka 1979; Allendorf et al. 2022), and contribute to population resilience by facilitating demographic stability and recolonization of vacant habitats (Brown and Kodric-Brown 1977).

While many species settle based on ecological or individual factors, cooperative breeders face additional social constraints that complicate dispersal and settlement decisions. In cooperative breeding species, individuals often delay dispersal beyond sexual maturity to help rear young within their natal group while awaiting breeding opportunities (Hamilton 1964; Koenig et al. 1992; Clutton-Brock et al. 2016). This behavior, known as philopatry, can increase indirect fitness through kin-selected benefits, although it comes at the cost of delayed direct reproduction (Clutton-Brock 2006). In contrast, dispersers may secure breeding opportunities more quickly, but at the risk of higher mortality (Boyd and Pletscher 1999; Clutton-Brock 2006; Kingma et al. 2016). In some cases, groups can even gain indirect fitness benefits by accepting unrelated immigrants, though such decisions are often shaped by complex tradeoffs involving group size, stability, and reproductive opportunities (Clutton-Brock et al. 2002; Koenig et al. 2016).

Unlike asocial species, the dynamics of social species are shaped not only by individual behaviors but also by the structure and interactions within the social group. In social groups, even the loss of a single member, such as a breeder, can destabilize hierarchies and alter dispersal patterns. Such disruptions may lead to increased dispersal, reduced group cohesion, and shifts in reproductive roles or helping behavior (Mech and Boitani 2003; Borg et al. 2015). These effects have been observed in multiple taxa such as in meerkats (*Suricata suricatta*); dominant breeder loss often leads to power struggles and group fission (Clutton-Brock et al. 2001); in acorn woodpeckers (*Melanerpes formicivorus*), breeder loss can destabilize cooperative coalitions and delay reproduction (Koenig et al. 2011), and in cooperatively breeding cichlids (*Neolamprologus pulcher*), helper fish may disperse or shift behavior following the loss of dominant individuals (Stiver et al. 2006). Such group-level consequences may influence not only the timing of dispersal but also whether immigrants settle in groups.

Immigration plays a key role in shaping group dynamics in cooperative breeding species, yet its social drivers are less understood. Immigration can alter group size, structure, and social hierarchies, which in turn affects reproductive success and cooperation (Jennions and Macdonald 1994; Fuller et al. 2003). For example, group size may influence disperser settlement into cooperative breeding groups. In African wild dogs (*Lycaon pictus*) larger groups can lead to improved reproductive success for dominant breeders within the group (Gusset and Macdonald 2010). As cooperative breeders with complex life histories, species such as gray wolves (*Canis lupus*) further complicate dispersal dynamics by switching groups multiple times over their lifespan (Mech and Boitani 2003). Wolves in the Northern Rocky Mountains, USA also rarely accept dispersers as helpers. Instead, they typically integrate adoptees as breeders within a group (Bassing et al. 2020; Ausband 2022a). This suggests a nuanced influence of kin selection in these populations (Hamilton 1964; Jennions and Macdonald 1994). Although wolf dispersal has been extensively studied (Jimenez et al. 2017; Mech 2020; Morales-González et al. 2021), the specific conditions that lead to immigrant settlement into established groups remain unclear (Morales-González et al. 2021).

How human pressures, such as harvest (eg, hunting and trapping), affect disperser settlement in cooperative breeding species has received little attention. Clarifying these patterns could improve predictions of population responses in species like gray wolves, as they can maintain population stability through compensatory dispersal when harvest rates remain < 29% (Fuller et al. 2003; Adams et al. 2008; Creel and Rotella 2010). However, this relies on consistent immigration from neighboring populations to offset mortality (Fuller et al. 2003). Bassing et al. (2020) found that immigration did not offset harvest mortality in wolf populations and suggested that the social structure of wolf groups can limit the compensatory effect of immigration. Even in stable populations, harvest can negatively impact wolf genetics, demographics, and social structures (Rick et al. 2017; Žunna et al. 2023). In kinship groups, human-caused mortality can create breeding vacancies or disrupt social structures, which may increase the benefits for subordinates to stay in their natal territory to wait for breeding opportunities (Ausband 2022a). At the same time, harvest can disrupt social dynamics and mating systems, leading to higher breeder turnover and increased juvenile dispersal.

Social and environmental complexities, along with variations in settlement patterns, shape wolf group dynamics (Jimenez et al. 2017; Ausband 2022a). In particular, social factors such as pack structure, kinship, and individual traits can shape settlement decisions (Mech 2020). Because few cooperative breeding species face intensive harvest, gray wolves in Idaho, USA offer an opportunity to study how such pressures influence dispersal patterns. The Idaho Department of Fish and Game set hunting and trapping seasons that created variation in annual harvest intensity over time, providing a natural experiment to examine how harvest affects group dynamics. While previous studies used experimental removals of individuals in small mammals and fish to study immigration (Hannon et al. 1985; Stiver et al. 2006), our research examines immigration into groups following potential removals due to harvest. We used long-term genetic data (2008 to 2020) from fecal samples to identify factors influencing disperser settlement.

We investigated how factors, including group size, breeder turnover, genetic relatedness, and harvest intensity affected the probability of successful immigration by a disperser. We predicted that higher genetic relatedness within groups would increase immigration due to inbreeding avoidance (Allendorf et al. 2022). We also predicted that smaller groups would be more likely to accept dispersers to grow group size because larger groups provide greater survival and breeding benefits (Jennions and Macdonald 1994; Heg et al. 2005; Gusset and Macdonald 2010; Ausband and Mitchell 2021). Additionally, we expected breeder turnover to influence immigration, specifically breeding male turnover, as they have higher dispersal tendencies (Jimenez et al. 2017; Morales-González et al. 2021). We also anticipated that increased wolf harvest would lead to more disperser immigration into groups due to additional breeding opportunities via harvested breeders. Next, we expected both harvest and group size to interact in shaping disperser settlement in groups because harvest reduces group size by removing individuals, which can disrupt social structure. Finally, we predicted that a global model would best explain successful immigration of dispersers, because all factors likely contribute to immigration outcomes.

## Methods

### Study area

**Figure 1:**
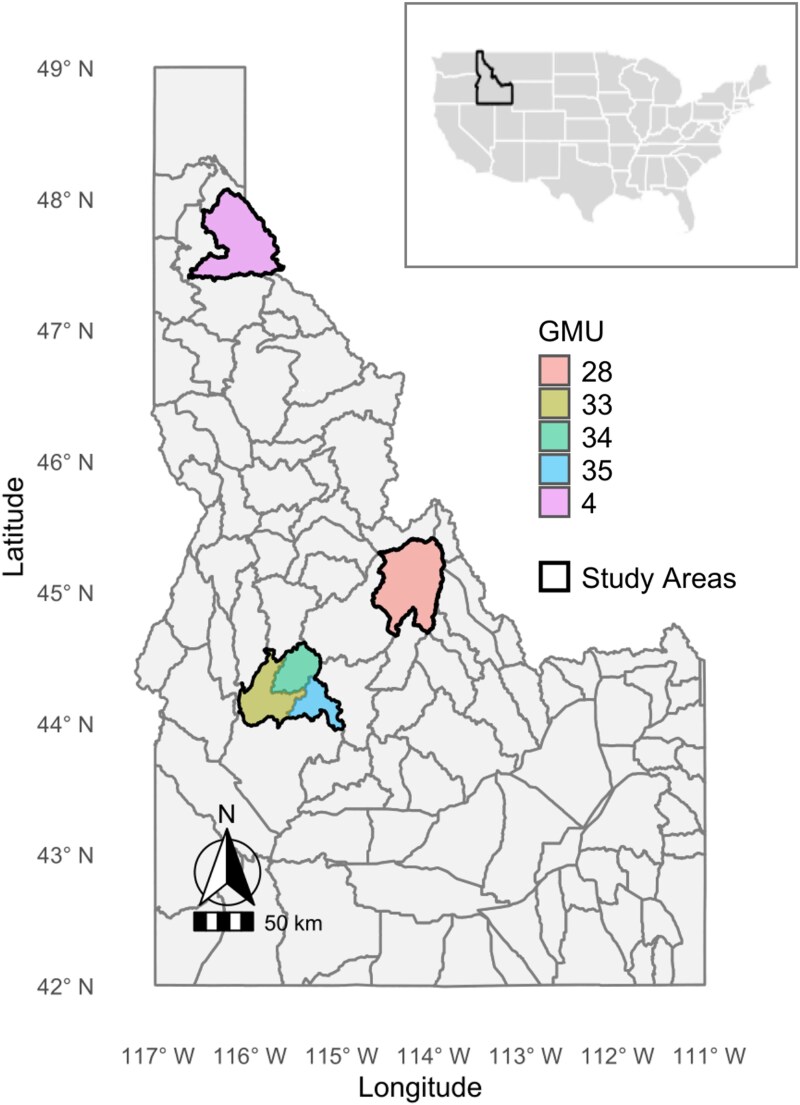
Map of Idaho, USA with highlighted Game Management Units (GMU) and outlined study areas that show 3 focal areas for gray wolf genetic sampling from 2008 to 2020.

Idaho, located west of the continental divide in the United States, features diverse topography and climates across its 216,632 km^2^. Elevations range from 217 to 3,859 m, with temperatures averaging −7 to 9 °C in winter (November to March) and 1 to 29 °C in summer (April to October). Our study combines statewide wolf harvest data with noninvasive genetic fecal sampling across 3 focal areas within 5 game management units (GMUs 4, 28, 33 to 35; Fig. 1), primarily on U.S. Forest Service land. Northern Idaho (GMU 4; 3,189 km^2^) has a maritime-influenced climate with warm, dry summers and cold, wet winters, receiving 64 to 127 cm of annual precipitation. Vegetation includes western hemlock (*Tsuga heterophylla*) at lower elevations and subalpine fir (*Abies lasiocarpa*) at higher elevations, with western red cedar (*Thuja plicata*) in moist areas. Douglas-fir (*Pseudotsuga menziesii*), ponderosa pine (*Pinus ponderosa*), and lodgepole pine (*P. contorta*) dominate drier sites. Logging activity is moderate to high. Central and eastern Idaho (GMU 28; 3,388 km^2^) transitions between coniferous forests, sagebrush steppe, and high desert. Annual precipitation ranges from 30.5 to 63.5 cm. This region includes the Salmon River and the Frank Church Wilderness Complex, the largest contiguous wilderness in the lower 48 states. Vegetation consists of sagebrush (*Artemisia tridentata*), lodgepole pine, and ponderosa pine. Southern Idaho (GMUs 33 to 35; 3,861 km^2^) spans parts of the Sawtooth, Payette, and Salmon-Challis National Forests. It includes arid canyons, agricultural land, and fragmented urban landscapes, with annual precipitation of 20.3 to 30.5 cm. The region supports diverse ecosystems across varied habitats. Across all 3 study areas, wolves prey on large and medium ungulates including moose (*Alces alces*), white-tailed deer (*Odocoileus virginianus*), mule deer (*O. hemionus*), and elk (*Cervus canadensis*).

### Field data collection

We used a long-term dataset (2008 to 2020) of fecal samples from gray wolves in Idaho and sampled packs repeatedly across years within the 3 study areas. Annually, field personnel gathered noninvasive genetic samples (wolf feces) in the 3 focal areas while monitoring 10 to 18 wolf groups. We followed field methods outlined in Ausband et al. (2010), which describes genetic sampling at pup-rearing, or rendezvous, sites, which are locations where groups of wolves congregate during the summer months before pups become more mobile. A predictive habitat model incorporating NDVI (greenness), roughness, and curvature identifies areas with high (≥70%) suitability for rendezvous sites (Ausband et al. 2010). We located these sites by using howl sets and wolf sign to pinpoint the center of wolf activity. Each year, we collected approximately 125 to 200 scat samples per group, consisting of roughly 60% adult and 40% pup samples. Adult (>2.5 cm diameter) and pup scats (<2.5 cm) were distinguished based on size of fecal samples within the rendezvous sites. Sampling efforts focused on these locations during the summer months (April to August), the optimal period for capturing an entire group before pups become more mobile.

### Laboratory methods

We extracted DNA from fecal samples collected from our 3 study areas using both Qiagen kits (Qiagen, Inc., Valencia, CA) and the Zymo Quick-DNA Fecal/Soil Microbe Miniprep Kit (Zymo Research, Irvine, CA) in a low-quality DNA facility at the University of Idaho Laboratory for Ecological, Evolutionary, and Conservation Genetics. Each extraction included a negative control to test for cross-contamination. Additionally, we performed a mitochondrial DNA species-identification test to identify target species and remove low-quality samples (de Barba et al. 2014). For tissue samples, we extracted DNA using Qiagen DNeasy Blood and Tissue kits (Qiagen Inc., Valencia, CA, USA) with negative controls.

We genotyped samples using a combination of 2 distinct polymerase chain reaction (PCR) multiplex assays, encompassing 18 nuclear microsatellite loci. We initially employed 10 microsatellite loci and primers to discern both individual identities and sex for all wolf samples. The additional 8 microsatellite loci were run exclusively on the highest-quality sample from each individual and were used to verify samples as needed (Stenglein et al. 2010, 2011; Stansbury et al. 2014). We separated PCR products by size using the Applied Biosystems 3130×1 capillary machine (Applied Biosystems Inc., Foster City, CA, USA) and called genotype peaks by eye with GENEMAPPER 5.0 (Applied Biosystems Inc.). We identified individual matches using Program Genalex (Peakall and Smouse 2012). We amplified all samples twice and excluded those with fewer than 5 successfully amplified loci. Samples yielding more than 5 loci underwent 1 to 3 additional replications. We determined heterozygotes by consensus from more than 2 independent PCR amplifications at each locus, while homozygote calls required more than 3 (Ausband et al. 2015). Every PCR amplification included both positive and negative controls. Following rarefaction results from Stenglein et al. (2011), we extracted DNA from 40 adult and 25 pup samples to achieve high detection probabilities, as Stenglein et al. found that detection of all individuals in a group asymptote at ∼65 samples. If a group had more than 2 individuals detected only once, we analyzed additional samples, if available, to obtain 10 additional consensus genotypes (Ausband et al. 2015). Additionally, genotypes of all single-capture individuals (<10% of all individuals) met the 95% reliability criteria (Miller et al. 2002). Analysis of 18 loci showed a very low probability of identity for siblings (ie, the chance that 2 individuals share the same genotype), ranging from 3.54 × 10^−4^ to 1.18 × 10^−3^ (Ausband et al. 2017). For more detailed information about laboratory techniques, consult Stenglein et al. (2010, 2011), and Stansbury et al. (2014).

### Pedigrees

**Figure 2:**
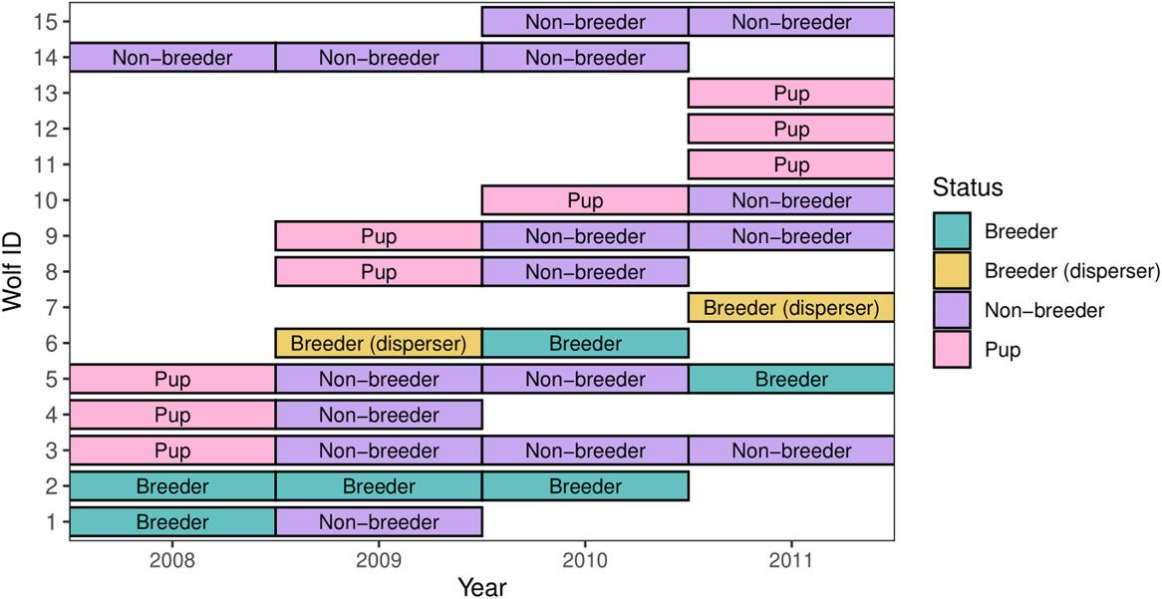
Example plot of 1 study group through time illustrating changes to group size and composition. Plot shows individual gray wolves and the status assigned per year, with each individual on the *Y* axis and surveyed years on the *X* axis for 1 pack in this dataset. Status can change between years. Breeder status indicates known parents of pups for that year. Disperser status indicates individuals not detected in earlier years within this pack. Nonbreeder status indicates helpers within the pack that did not breed that year.

After collecting wolf fecal samples and extracting DNA, we used genotypes of individual wolves to construct pedigrees to determine paternity and maternity. We considered all adults as potential parents and all sampled pups as potential offspring each year. We constructed familial pedigrees from rendezvous site data using maximum-likelihood analyses in Program COLONY, version 2.0.5.5 (Jones and Wang 2010). We initially calculated allele frequencies for each year using Program COANCESTRY version 1.0.1.5 (Wang 2011) and imported the results into Program COLONY for pedigree analysis. COLONY employs a maximum-likelihood approach to estimate the probabilistic parent-offspring relationships between individuals. We permitted polygamy in both sexes and set an allelic dropout rate of 0.01, with other genetic error rates, including mutations, also set to 0.01. From COLONY, we generated pedigrees for each wolf group. These analyses determined age classes and familial roles from genetic relationships. We classified breeders as adults determined through pedigree analyses to be parents of sampled pups and assigned pups to genetically related parents. All other samples confirmed as adults in the field and not identified as breeders were considered nonbreeding helpers for each group per year (Fig. 2).

### Disperser identification

We identified dispersing wolves using the genetic pedigrees for each group. We defined dispersers as unrelated individuals within a group who were absent during the previous survey year but present during the following survey year (Stansbury et al. 2016). Because most wolf dispersal in North America occurs during the fall–winter period (Ballard et al. 1987; Boyd and Pletscher 1999; Jimenez et al. 2017), we assigned the dispersal year to the previous calendar year, assuming the disperser left their natal pack after our summer detection period. We assigned the settlement year to the calendar year when we first detected the disperser within a new group, indicating when the disperser immigrated into the group. This approach accounts for dispersers who joined a group during fall or winter but were not observed until the next survey year.

### Breeder turnover and group size

Pedigrees provided estimates of group size, identified breeder turnover, and determined when a disperser from outside the group filled a breeding position. We classified breeder turnover when a male or female genetically confirmed as a breeder with pups in 1 year was replaced by a new breeder in the following year (see Fig. 2 eg of breeder turnover). Only cases with 2 consecutive years of data confirming a change in a breeding individual, based on parentage of pups, were considered turnover events. Dispersers often joined a group and became breeders within their first or second year (Ausband et al. 2017), causing a change in breeding individuals and constituting a turnover event. This definition captures changes in breeders within a pack and may include instances following vacancies created by harvested wolves, although such vacancies were not directly separated in this study. We defined group size as the number of adult wolves in a group during the settlement year. Parentage results from COLONY and consensus genotypes provided annual estimates of adult numbers in each group per year. For groups with a disperser, we removed that individual from the group size count. Settlement year reflects group composition after the fall and winter period, when human-caused and natural mortality is highest and juveniles most often emigrate (Jimenez et al. 2017). We excluded pups of the year because they were not alive during the previous fall and winter dispersal period.

### Genetic relatedness

To estimate genetic relatedness among individuals in the group, we used the Trio ML relatedness coefficient in Program COANCESTRY (Wang 2011) which compares triads of individuals to create a probability value of relationships. We calculated the mean genetic relatedness values between all adult wolves in the group dispersers joined from the dispersal year and excluded dispersers in the calculation. In this context, mean relatedness may predict the propensity for a disperser to settle into a group.

### Wolf harvest

In Idaho, hunters and trappers who harvest a wolf are required to check and report their harvest within 10 days, and they must present the skull and hide to an IDFG office. From 2009, and 2011 to 2020, state personnel removed a premolar tooth, used for cementum aging (conducted by Matson's Laboratory, Manhattan, MT, USA), as well as collected biological tissue samples. To estimate the wolf harvest intensity at the population level, we divided the total number of wolves harvested in each study area by its respective area (in km^2^), scaled to 1,000 km^2^ for comparability across GMUs. Because the southern study area comprises 3 GMUs (33, 34, 35), we summed their respective number of harvested wolves and GMU areas to obtain a total estimate. We assigned harvest intensity based on the dispersal year because it encompasses the harvest season during dispersal of that individual (fall–early winter) and potential vacancies created during this time in the group they joined.

### Statistical analysis

To examine factors predicting whether a wolf successfully immigrates into a group, we fit a generalized linear model (GLM) with a binomial distribution and a logit link function using program R (R core Team 2025). The response variable was whether a wolf pack accepted a disperser (binary: 0 = no, 1 = yes) in each survey year. Predictor variables included female and male breeder turnover, mean genetic relatedness, wolf harvest, and group size. All predictors were standardized using *z*-scores for comparability.

We began by fitting a GLM with only the standardized fixed effects. Next, we fit a GLM that included an interaction between standardized group size and standardized wolf harvest to assess how different harvest intensities influence the probability of immigration, depending on group size. We considered a generalized linear mixed-effects model (GLMM) with pack as a random effect, but it failed to converge, which produced unreliable results. We therefore used a GLM, which fit the data well. We evaluated candidate models using Akaike Information Criterion (AIC; Akaike 1974). We also assessed model fit using the DHARMa R package (Hartig 2024), which creates standardized, simulation-based residuals for generalized linear models that can be interpreted like traditional residuals. Finally, we evaluated model fit using the Area under the Curve (AUC) and McFadden's *R*^2^ for the top model.

## Results

We analyzed data from 105 wolf groups between 2008 and 2020, with some packs represented in multiple years (mean = 4.78 years sampled per pack, SD = 3.25), all within the 3 study areas. We identified 26 dispersers, and 30% of groups accepted a disperser. Group size averaged 5.97 wolves (range: 1 to 14), while wolf harvest averaged 4.1 wolves per 1,000 km^2^ (range: 1 to 15.4). The average genetic relatedness value was 0.33 (range: 0.00 to 0.50). There were 33 male and 28 female breeder turnover events. Of the 26 dispersers who joined packs, 85% (22 individuals) became confirmed breeders within their first or second year: 16 in their first year and 6 in their second. Four never bred during the study, 3 of which were detected only in their year of immigration.

**Figure 3:**
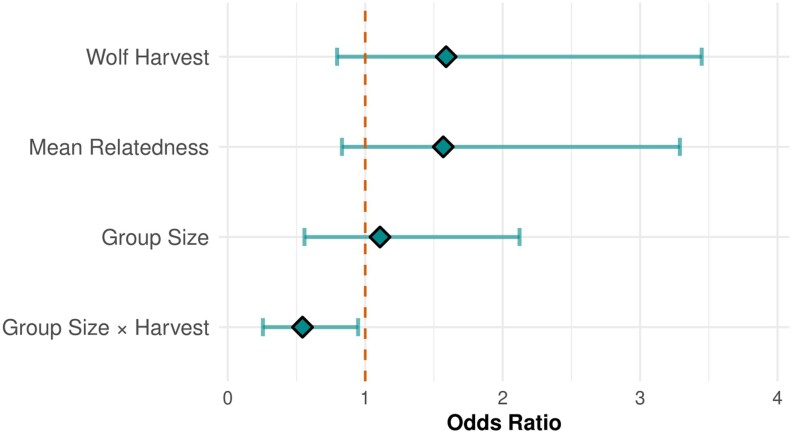
Forest plot of odds ratio for variables in the top model predicting factors that predict the probability of disperser immigration in Idaho, USA, 2008 to 2020. Variables are on standardized scales, with all other variables held at their mean values. Confidence intervals are displayed at the 95% level. Sex effects are not displayed.

For model selection, the GLM with an interaction between group size and wolf harvest had the lowest AIC (Akaike 1974) and the highest model weight, making it the best-supported model for predicting disperser immigration (Table 1). We assessed model fit using DHARMa standardized residuals, which indicated that the top model fit well: scaled residuals distributed uniformly (uniformity test *P* = 0.76), no extreme residuals occurred (outlier test *P* = 1), and residual variance aligned with model assumptions (dispersion test *P* = 0.62). Residual diagnostics showed no strong deviations from model assumptions, suggesting that re-sampling did not strongly bias the estimates. This model had an AUC score of 0.86, suggesting good predictability, and a McFadden's *R*^2^ value of 0.30, indicating a reasonable fit to the data compared with the null model. Among the predictor variables (Table 2, Fig. 3), male breeder turnover had a statistically significant positive effect on the probability of a group accepting a disperser (*β* = 2.08, SE = 0.64, 95% CI −0.87 to 3.40, *P* = 0.001). In contrast, female breeder turnover (*β* = 0.97, SE = 0.64, *P* = 0.12) and mean group relatedness (*β* = 0.45, SE = 0.34, *P* = 0.19) did not have significant effects on disperser immigration. Neither group size nor wolf harvest had a significant main effect on the probability of a disperser settling in a group. However, we found a marginally significant crossover interaction between the 2 (*β* = −0.61, SE = 0.32, *P* = 0.058). The negative sign of the interaction suggests that the relationship between wolf group size and disperser immigration depends on harvest intensity. In contrast, other variables, such as male breeder turnover (*β* = 2.08), had a stronger effect on disperser settlement.

**Table 1:** Candidate models for predicting successful immigration of dispersing gray wolves in Idaho, USA, 2008 to 2020.

Model	*K*	LL	AIC	ΔAIC	AIC*w_i_*
Genetic relatedness + Group size + Wolf harvest + Male Turnover + Female Turnover + Group size × Wolf Harvest ^a^	7	−36.7	87.4	0	0.73
Genetic relatedness + Group size + Wolf harvest + Male Turnover + Female Turnover	6	−39.1	90.1	2.69	0.19
Male Turnover	2	−44.0	92.0	4.63	0.07
Female Turnover	2	−47.4	98.8	11.375	0.00
Null	1	−52.7	107.4	20.00	0.00
Genetic relatedness	2	−61.6	127.2	39.84	0.00
Wolf harvest	2	−74.7	153.3	65.91	0.00
Group size	2	−77.8	159.7	72.27	0.00

Models include ecological and biological predictors such as mean genetic relatedness, group size, wolf harvest, male breeder turnover, female breeder turnover, and the interaction between group size and wolf harvest.

^a^Indicates most supported model, AUC = 0.86, McFadden's *R*^2^ = 0.30

**Table 2:** Covariates from the most supported model predicting gray wolf immigration of dispersers into groups in relation to mean genetic relatedness, group size, wolf harvest, male breeder turnover, female breeder turnover, and the interaction between group size and wolf harvest in Idaho, USA, 2008 to 2020.

Covariate	*β*	SE	*P*
Intercept	−2.37	0.51	NA
Genetic relatedness	0.45	0.34	0.19
Group size	0.10	0.33	0.75
Wolf harvest	0.46	0.37	0.21
Male turnover	2.08	0.64	0.001
Female turnover	0.97	0.64	0.12
Group size × Wolf harvest	−0.61	0.32	0.06

The table provides estimated coefficients (*β*), standard errors (SE), and *P*-values (*P*) for each covariate.

## Discussion

Our results indicate that successful immigration of dispersers depends on both social and environmental contexts. Male breeder turnover and the interaction between group size and harvest pressure significantly influenced immigration into groups, suggesting that socially-informed dispersal plays a key role in shaping group composition of cooperative breeders under varying ecological conditions. Specifically, immigration increased following male breeder turnover, indicating that the loss and replacement of a male breeder may open social space for outside individuals. This process likely involves socially-informed decision-making, where both dispersers and group members respond to cues such as breeder turnover (Cozzi et al. 2018). Male dispersers may exploit temporary breeding opportunities created by such turnover. Additionally, the interaction between group size and harvest pressure suggests that external forces, such as human-caused mortality, alter how groups respond to incoming dispersers.

**Figure 4:**
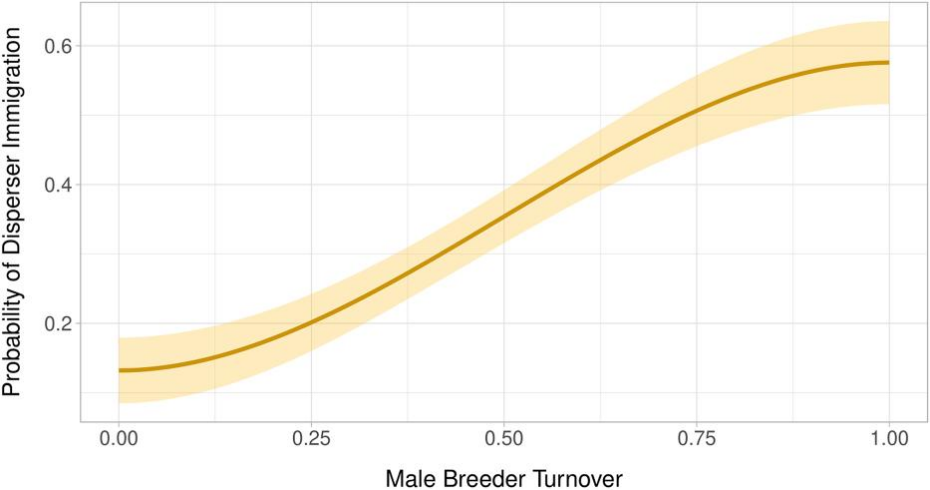
Effect plot for the significant variable in the top model predicting factors that predict the probability of a disperser immigration in Idaho, USA, 2008 to 2020. All other variables are held at their mean values in the plot. Confidence intervals are displayed at the 95% level.

Breeder turnover (Fig. 4) illustrates how sociality shapes settlement patterns of dispersers, as dispersers were more likely to join a group following the loss of a breeding male. Turnover creates breeding opportunities that dispersers can exploit (Ausband 2022a), and as social instability increases, groups may become more receptive to new members. This may prompt a shift from kin-based cooperation to group augmentation (Kokko et al. 2001). Previous studies on other social group dynamics, such as cichlid fish (*Neolamprologus pulcher*) and prairie dogs (*Cynomys ludovicianus, C. gunnisoni, C. parvidens*), show that breeder turnover can disrupt social cohesion and create opportunities for dispersal (Stiver et al. 2006; Hoogland 2013). These disruptions may stem from shifts in social hierarchy, reduced competition from former breeders, or a need to replenish group members. Losing reproductive individuals in complex social species can destabilize group structure and slow population growth, but rapid breeder replacement may minimize social disruption (Borg et al. 2015). For example, African wild dog packs experiencing breeder turnover are more likely to integrate new members than those with stable breeding pairs (Creel and Creel 2002). Similarly, gray wolves show a high rate of group dissolution following breeder loss, underscoring the importance of group expansion and social stability. These patterns suggest that filling breeder vacancies provides direct fitness benefits that stabilize cooperative groups.

Previous research in this study system has explored the social and genetic dynamics of cooperatively breeding carnivores, but this study focuses on how group structure and context drive disperser settlement. The strong effect of male breeder turnover aligns with established patterns of male-biased dispersal, where males more often leave natal territories and secure breeding roles through dispersal, while females tend to inherit positions within natal groups (Ausband 2022a). Additionally, male-biased litters likely reinforce this dispersal pattern (Ausband 2022b). The influence of male turnover on successful immigration of dispersers highlights the central role of males in group stability, persistence, and broader dispersal dynamics in this system. This period of social reorganization can increase group fluidity and create opportunities for new individuals to integrate (Packard 2003; VonHoldt et al. 2008; Koenig et al. 2016). Furthermore, we found that harvest pressure changes how group size affects disperser acceptance, demonstrating that harvest influences not only survival but also social decisions.

**Figure 5:**
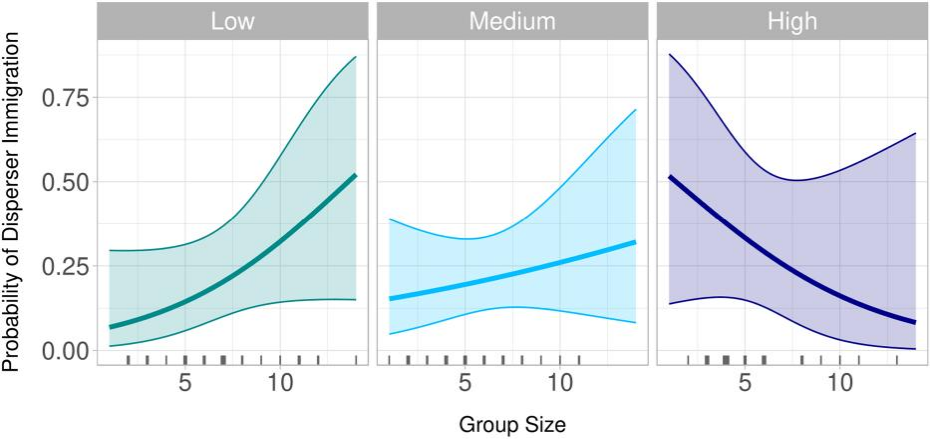
Effect plots for the interaction between harvest and group size in the top model predicting the probability of disperser immigration in Idaho, USA, from 2008 to 2020. Harvest was categorized into low, medium, and high levels. Confidence intervals are displayed at the 95% level. Tick marks along the *x*-axis show sample distribution. Larger ticks indicate more samples at a given value, and smaller ticks indicate fewer samples.

The relationship between group size, harvest pressure, and immigration of dispersers is complex (Fig. 5). Under low harvest pressure, larger groups may be more willing to accept dispersers, possibly due to their capacity to absorb new members and benefit from added benefits of group size such as experience advantages such as improved hunting, territory defense, and pup-rearing (Mech and Boitani 2003; Borg et al. 2015). In contrast, under high harvest pressure, larger groups may prioritize internal recruitment to preserve social cohesion, reflecting a shift in strategy driven by kin selection (Hamilton 1964). The increased likelihood of larger groups predicting immigration could be a strategy to maintain large group cohesion and resilience (Mech and Boitani 2003; Koenig et al. 2016). Furthermore, we found under high harvest pressure, the relationship switches. In this circumstance, smaller groups are more likely to have dispersers immigrate, whereas larger groups as less likely. These smaller groups may find group augmentation essential for maintaining group stability (Kokko et al. 2001), inbreeding avoidance and group persistence (Sparkman et al. 2012). Disperser settlement may be a necessary strategy for survival and cohesion in small groups under high harvest pressure. In this case, larger sized groups may maintain social structure by relying on internal recruitment, such as pup recruitment or female philopatry to fill breeding positions, rather than accepting outsiders (Hamilton 1964; Clutton-Brock 2006).

Our research did not find significant effects for several variables expected to influence successful immigration of dispersers. Genetic relatedness did not predict immigration outcomes, which suggests flexibility in cooperative breeding systems (Ausband 2018). This aligns with carnivore systems where reciprocity can enhance opportunities for successful immigration by promoting integration of nonkin that may provide cooperative benefits over time, and where immigration by unrelated individuals can lower inbreeding risk within highly related groups. Female breeder turnover also did not affect immigration outcomes. This likely reflects the small proportion of female dispersers detected in our data and the tendency for females to remain philopatric and queue for breeding opportunities within natal groups (Mech and Boitani 2003; Vonholdt et al. 2008; Ausband 2022a). Cooperative breeding frameworks suggest female philopatry can strengthen sex-biased dispersal (Perrin and Goudet 2001). These results indicate that settlement success of dispersers in this system reflect individual opportunities shaped by social and environmental contexts, rather than genetic relatedness or female turnover alone.

Unlike studies focused on individual dispersal decisions, our research examines conditions that support or limit successful immigration under shifting ecological and social conditions. Our research linked external pressures (harvest) and internal events (breeder turnover) to disperser integration to reveal how both direct and indirect social cues influence the perception of dispersers within the social landscape (Bonte et al. 2012). Our results suggest that dispersal may be socially-informed, with individuals responding to cues such as breeder turnover, group size, and social stability during dispersal decisions (Clobert et al. 2009; Cozzi et al. 2018). We tracked social and genetic relationships using genetic relatedness and breeder turnover across time. This approach requires intensive effort and may not be feasible in all studies of group-living mammals. In our case, because most wolf dispersal occurs in the fall and winter, we assigned dispersal year to the previous calendar year. However, some individuals may have dispersed before the summer sampling period, which could introduce mismatches between covariates and actual dispersal timing. By leveraging a broad scale experiment created by harvest, our study examined how removal of individuals influences disperser settlement dynamics in cooperative breeders and contributes to broader conceptual frameworks. Driscoll et al. (2014) emphasized persistent limitations in dispersal ecology, with nearly half of reviewed studies identifying gaps that limit ecological inference and conservation outcomes. Clarifying how these social cues shape settlement can improve conservation and management strategies.

## Supplementary Material

arag013_Supplementary_Data

## Data Availability

Analyses reported in this article can be reproduced using the data provided by Author (2026).
